# Angiogenic effect of the aqueous extract of *Cynodon dactylon* on human umbilical vein endothelial cells and granulation tissue in rat

**DOI:** 10.1186/s40199-015-0093-x

**Published:** 2015-01-29

**Authors:** Hamid Soraya, Milad Moloudizargari, Shahin Aghajanshakeri, Soheil Javaherypour, Aram Mokarizadeh, Sanaz Hamedeyazdan, Hadi Esmaeli Gouvarchin Ghaleh, Peyman Mikaili, Alireza Garjani

**Affiliations:** Department of Pharmacology, Faculty of Pharmacy, Urmia University of Medical Sciences, Urmia, Iran; Student of Veterinary Medicine, Faculty of Veterinary Medicine, Urmia University, Urmia, Iran; Department of Immunology, Faculty of Medicine, and Cellular & Molecular Research Center, Kurdistan University of Medical Sciences, Sanandaj, Iran; Department of Pharmacognosy, Faculty of Pharmacy, Tabriz University of Medical Sciences, Tabriz, Iran; Department of Microbiology, Faculty of Veterinary Medicine, Urmia University, Urmia, Iran; Department of Pharmacology & Toxicology, Faculty of Pharmacy, Tabriz University of Medical Sciences, Tabriz, Iran

**Keywords:** Cynodon dactylon, Angiogenesis, Air pouch, HUVECs, VEGF

## Abstract

**Background:**

*Cynodon dactylon*, a valuable medicinal plant, is widely used in Iranian folk medicine for the treatment of various cardiovascular diseases such as heart failure and atherosclerosis. Moreover, its anti-diabetic, anti-cancer and anti-microbial properties have been also reported. Concerning the critical role of angiogenesis in the incidence and progression of tumors and also its protective role in cardiovascular diseases, we investigated the effects of the aqueous extract prepared from the rhizomes of *C. dactylon* on vascular endothelial growth factor (VEGF) expressions in Human Umbilical Vein Endothelial Cells (HUVECs) and also on angiogenesis in carrageenan induced air-pouch model in rats.

**Methods:**

In the air-pouch model, carrageenan was injected into an air-pouch on the back of the rats and following an IV injection of carmine red dye on day 6, granulation tissue was processed for the assessment of the dye content. Furthermore, in an *in vitro* study, angiogenic property of the extract was assessed through its effect on VEGF expression in HUVECs.

**Results:**

Oral administration of 400 mg/kg/day of the extract significantly increased angiogenesis (p < 0.05) and markedly decreased neutrophil (p < 0.05) and total leukocyte infiltration (p < 0.001) into the granulation tissues. Moreover, the extract increased the expression of total VEGF in HUVECs at a concentration of (100 μl/ml).

**Conclusion:**

The present study showed that the aqueous extract of *C. dactylon* promotes angiogenesis probably through stimulating VEGF expression.

## Background

Recently, medicinal plants have been largely considered as the harmless alternate to synthetic drugs, especially due to their more safety and less side effects as compared to the chemical drugs. *Cynodon dactylon*, also known as Bermuda grass, is a perennial grass native to the warm temperate and tropical regions [1]. In North West of Iran, *C. dactylon* is known as “Chayer” and the aqueous extract of its rhizomes is widely used in the treatment of cardiovascular disorders such as atherosclerosis and heart failure due to its hypolipidemic and cardiac tonic effects [2,3]. However, despite the presence of several reports on the anti-diabetic, anti-microbial [4], hypolipidemic [5], hepatoprotective [6], anti-emetic and anti-inflammatory [7] properties of *C. dactylon*, the probable role of angiogenesis as the mechanism involved in cardioprotective effect of the plant has remained largely elusive.

Angiogenesis, the formation of new blood vessels from pre-existing capillaries, plays an important role in many physiologic and also pathologic processes including cancer, ischemic heart diseases and chronic inflammation [8,9]. Moreover, since the therapeutic interference with angiogenesis offers a valuable tool for clinical application in several pathological conditions, much attention has been paid on medications or compounds that alter the gene expression profile of vascular endothelial cell growth factor (VEGF) and fibroblast growth factors (FGFs) as two key pro-angiogenic molecules [10]. In some cases such as heart failure or cardiac ischemia-reperfusion, stimulation of angiogenesis is beneficial and can be considered as a target to improve the disease condition, however in several other diseases such as cancer, atherosclerosis, rheumatoid arthritis and diabetic retinopathy, excessive angiogenesis is part of the pathology and progression of the disease [9-11]. Therefore, stimulation or inhibition of angiogenesis to reach therapeutic purpose is dependent on the type of the disease.

Marappan and Subramaniyan [12] assessed anti-tumor effects of the methanolic extract of *C. dactylon* leaves against ascetic lymphoma (ELA) in Swiss albino mice. The results showed that the plant possesses significant anti-tumor effects [12]. Another study by Krishramoorthy and Ashwini [13] also reported anti-cancer effects of *C. dactylon* in Swiss albino mice. The hydroalcoholic extract from rhizomes of *C. dactylon* has been shown to have strong protective effect on right heart failure in rats, in part by improving cardiac function and increasing the contractile force [2]. In another study this effect was also attributed to its anti-arrhythmic activity [14]. The plant also increased heart beat rate in a study on zebrafish with a potency greater than that of betamethasone [15].

As stated above, *C. dactylon* has been proven to be an effective cardiovascular agent by several studies; however almost none of the studies have precisely investigated the underlying mechanisms through which this plant exerts its effects on the cardiovascular system. The authors speculate that such beneficial effects of *C. dactylon* might be at least partly associated with its probable effects on angiogenesis. Since there have been no studies conducted so far on the possible effects of *C. dactylon* on angiogenesis, the present study was carried out to investigate the possible angiogenic activity of the plant in human umbilical vein endothelial cells (HUVECs) and also in an air-pouch model in rats.

## Materials and methods

### Extract preparation

*C. dactylon* was purchased from a traditional herbal market and the genus and species was authenticated at the Herbarium of Botany, Faculty of Pharmacy, Urmia, Iran. The rhizomes of the plant were dried in shade and coarsely ground to powder using an automatic grinder. 200 g of the powder was mixed in 2 L of distilled water and placed on a magnet stirrer at a temperature of 50°C for three days. The mixture was then filtered three times using the Wattman’s paper. The solution was finally evaporated to dryness for 12 hrs at 70°C. The total amount of the crude extract obtained was 34 g. The extract was diluted with water in order to be given orally by gavage needle.

### Preliminary phytochemical screening

Qualitative phytochemical analysis of *C. dactylon* aqueous extract was conducted following the standard procedures as described by Harborne [16], Sofowora [17] and Trease and Evans [18].

### Alkaloids

Crude extract was mixed with 2 ml of 1% HCl and heated gently. Mayer’s reagent was then added to the mixture. Turbidity of the resulting precipitate is as evidence for the presence of alkaloids.

### Anthocyanins

2 ml of aqueous extract was added to 2 ml of 2 N HCl and NH_3_. Manifestation of pink-red turning blue-violet indicates the presence of anthocyanins.

### Coumarins

3 ml of 10% NaOH was added to 2 ml of aqueous extract, formation of yellow color indicates the presence of coumarins.

### Flavonoids

Crude extract was mixed with few fragments of magnesium ribbon and concentrated HCl was added drop wise. Appearance of pink scarlet color after few minutes indicates the presence of flavonoids (Shinoda test).

### Saponins

Crude extract was mixed with 5 ml of distilled water in a test tube and shaken vigorously to obtain a stable persistent froth. The frothing is then mixed with 3 drops of olive oil and for the formation of emulsion which indicates the presence of saponins.

### Tannins

Crude extract was mixed with 2 ml of 2% solution of FeCl_3_. Observed blue-green or black coloration indicates the presence of tannins.

#### Assay for in vitro antioxidant activity

The free radical scavenging capacity of the extract was measured from the bleaching of the purple-colored methanolic solution of 2,2-diphenyl-1-picrylhydrazyl)DPPH), a routinely practiced material for the assessment of antiradical properties of different compounds [19]. The stock concentration of the *C. dactylon* aqueous extract (1 mg/mL) was prepared followed by dilution to reach for concentrations 5 × 10^−1^, 2.5 × 10^−1^, 1.25 × 10^−1^, 6.25 × 10^−2^, 3.13 × 10^−2^ and 1.56 × 10^−2^ mg/mL of the extract. The acquired concentrations in the same volumes of 2 mL were added to 2 mL of a 0.08 % of DPPH solution [20-22]. Later than a 30 min of incubation at 30°C, the absorbance of each solution was read against a blank sample at 517 nm (Shimadzu 2100 spectrophotometer - Japan). The average absorption value was noted for each sample after the test was carried out in triplicate. Besides, as the positive control the same procedure was gone over with quercetin. The inhibition percentage of DPPH free radicals of by the aqueous extract was calculated as follows:

$$ \mathrm{R}\ \left(\%\right) = 100 \times \left[\left(\mathrm{A}\ \mathrm{blank}\ \hbox{--}\ \mathrm{A}\ \mathrm{sample}\right)/\mathrm{A}\ \mathrm{blank}\right] $$

Herein, “A blank” stands for the absorbance value of the control reaction and “A sample” is the absorbance value for each sample. Additionally, RC_50_ value, the concentration of the extract reducing 50% of the DPPH free radicals, was calculated from the graph of inhibition percentages versus concentrations of *C. dactylon* extract in mg/mL.

#### Assay for total phenolics content

Total phenolic constituents of the *C. dactylon* aqueous extract was verified by assigning Folin-Ciocalteu reagent and gallic acid as the standard compound for phenolics, the same procedure as given in the literature [23-26]. Briefly, 0.5 mL of the extract was mixed with 5 mL of Folin-Ciocalteu reagent (10% v/v in distilled water) with 4 mL of 1 M aqueous Na_2_CO_3_ after 5 min and the mixture was allowed to stand for 15 min with intermittent shaking. The absorbance of the blue color produced by the reaction was measured using a UV/visible spectrophotometer (Shimadzu 2100 - Japan) at 765 nm. The standard curve was prepared using 25–300 μg/mL solutions of gallic acid in methanol: water (50:50, v/v). Eventually, the value for total phenol content of the *C. dactylon* extract was represented in terms of gallic acid equivalent which is a common reference compound.

#### Animals

Albino Wistar rats (220-250 g) were used in this study. Rats were housed at constant temperature (20 ± 1.8°c) and relative humidity (50 ± 10%) in standard polypropylene cages, eight per cage, under a 12 L:12D schedule and were allowed food and water freely. This study was performed in accordance with the Guide for the Care and Use of Laboratory Animals of Urmia University of Medical Sciences, Urmia-Iran.

#### In vivo angiogenesis assay

Rats were divided into 5 groups consisting 8 rats each. Rats in group 1 (carrageenan) received intra-pouch injection of carrageenan and normal saline as vehicle (0.5 ml) was given orally. Rats in group 2 received intra-pouch injection of carrageenan and intraperitoneal injection of dexamethasone. Rats in group 3 to 5 received intra-pouch injection of carrageenan and cynodon dactylon extract was given orally at doses 100, 200 and 400 mg/kg/day. For analyzing the effects of the aqueous extract of *C. dactylon* on *in vivo* angiogenesis, the air-pouch model described by Gosh *et al*. [8] was used with minor modifications. Briefly, rats (n = 8) were lightly anesthetized with diethyl ether, the back was shaved and then swabbed with 70% ethanol. Subsequently, 8 ml of sterile air was injected subcutaneously on the back of the animals to make an air-pouch oval in shape. Twenty four hours later, 4 ml of a 1% (w/v) solution of carrageenan (Sigma Co; USA) in saline was injected into the air-pouch under light diethyl ether anesthesia. The carrageenan solution had been sterilized by autoclaving at 121°C for 15 min and supplemented with penicillin G potassium and streptomycin sulfate (JaberEbne – e- Hayyan, Iran) (0.1 mg/ml of the solution) after cooling to 40–45°C. The aqueous extract of *C. dactylon* was administered orally at doses of 100, 200 and 400 mg/kg/day and dexamethasone as a standard anti-inflammatory agent (10 mg/kg/day) was injected intraperitoneally, a day before and for 6 days after carrageenan injection.

#### Determination of angiogenesis in granulation tissue

Six days after carrageenan injection, measurement of the angiogenesis in the granulation tissue was carried out using carmine dye, as an indicator of angiogenesis, according to the methods described by *Gosh et al*. [8] with slight modification. Briefly, the rats were anesthetized by intraperitoneal injection of a mixture of ketamine (60 mg/kg) and xylazine (10 mg/kg). Then 3 ml of 5% (w/v) carmine dye (Sigma Co; USA) in 5% (w/v) gelatin (Sigma Co; USA) in saline at 37°C was injected into the jugular vein of each rat. The carcasses were chilled on ice for 3 hrs and then the entire granulation tissue was dissected and weighted. After being washed with PBS (PH 7.4) the whole granulation tissue was homogenized in two volumes of 0.5 mM sodium hydroxide using a T25 basic homogenizer (IKA labortechnik, Gremany) for 4 min at 10000 *g* on an ice bed. The tissue homogenate was centrifuged at 3000 *g* and 4°C for 45 min and 500 μl of the supernatant was diluted 2-fold with 0.5 mM sodium hydroxide and centrifuged again. Then 100 μl of the supernatant was diluted with 900 μl of 0.5 mM sodium hydroxide and the carmine dye content was assessed spectrophotometrically at a wavelength of 490 nm. For histopathological visualization of the granulation tissues, the tissues were fixed in 10% (v/v) formalin in PBS for 48 hrs at 4°C. The samples were dehydrated by continuous immersion in 70% (v/v) ethanol for 48 hrs, 90% (v/v) ethanol for 48 hrs, and pure ethanol for 48 hrs. After dehydration, the samples were cleared by their immersion in the cedarwood oil (Sigma Co; USA) for 14 days. Retention of carmine dye within the vascular bed was observed with a light microscope (40× magnification).

#### Determination of pouch fluid accumulation, granulation tissue weight, total leukocyte infiltration along with lymphocyte and neutrophil percentage in the pouch exudates

Six days after the carrageenan injection, total pouch fluid was collected, the volume was measured and the entire granulation tissue was dissected and weighted. The total leukocyte count was determined in a neubauer chamber and the differential cell count was determined by microscopic counting of Gimsa stained slides.

#### In vitro angiogenesis assay

The expression changes in cytoplasmic and surface levels of VEGF in PBS or extract treated HUVECs were determined using a PAS flow cytometer (Partec GmbH, Germany). Briefly, human umbilical vein endothelial cells (HUVECs) were cultured at 37°C and 5% CO2 in low-glucose Dulbecco’s Modified Eagle’s Medium (LG-DMEM) supplemented by Supplement Mix (PromoCell) and10% fetal bovine serum (FBS). At second passage cells were treated with PBS (100 μl/ml) and *C. dactylon* extract (100 μl/ml). After 12 hrs using trypsin/EDTA (Ethylenediaminetetraacetic acid) solution (0.25%), cells were detached and then washed twice in PBS. The collected cells were permeablized with 0.1% PBS-Tween for 20 min. After incubation in 1× PBS/10% normal goat serum/0.3 M glycine to block non-specific interactions, unconjugated rabbit anti human VEGF antibody (abcam) was added. Subsequently, staining was performed using secondary goat anti-rabbit IgG PE antibody. The total 20000 events for each sample were acquired. Flow max software was used for data analysis.

#### Statistical analysis

Data were presented as mean ± standard error of the mean (SEM) and were analysed using one-way-ANOVA to make comparisons between the groups. If the ANOVA analysis indicated significant differences, Student–Newman–Keuls post test was performed to compare the mean values between the treatment groups and the control group. Differences between groups were considered significant at P < 0.05.

## Results

### Phytochemical analysis

Phytochemical compounds such as alkaloids, anthocyanins, coumarins, flavonoids, saponins, tannins and phenolic compounds were screened in the *C. dactylon* aqueous extract. Availability of these compounds, important secondary metabolites, has been tabulated in Table 1. Among the selected compounds alkaloids, coumarins, saponins and some phenolics, were present in the plant which could be responsible for the medicinal values of the respective plant. Moreover, the amount of total phenolic compounds in the *C. dactylon* aqueous extract established through Folin Ciocalteu method was calculated as gallic acid equivalent. The equation [Sample absorbance = 0.0067 × gallic acid (μg) + 0.0132, (R^2^: 0.987)] achieved by the standard gallic acid graph was applied in calculation of the phenolic compounds concentration. Subsequently, the content for *C. dactylon* total phenolics showed the value 39.82 mg of gallic acid equivalent in g of plant extract (Table 1). The findings of the antioxidant activity for *C. dactylon* aqueous extract, accomplished by the DPPH method, revealed sensible values *in vitro*. Regarding the results for the DPPH radical scavenging antioxidant assay, *C. dactylon* extract exhibited pleasant antioxidant activity with RC_50_ values of 0.70 mg/ml for the extract and 3 μg/mL for the control quercetin (Table 1). Quantitative phytochemical analysis revealed that the plant contained phenolic compounds a class of phytochemicals that could be responsible for the antioxidant and free radical scavenging effect of the plant material.

**Table 1:** **Phytochemical analysis**, **DPPH radical scavenging capacity and total phenols content of** ***C. dactylon*** **aqueous rhizomes extract**

	**Alkaloids**	**Anthocyanins**	**Coumarins**	**Flavonoids**	**Saponins**	**Tannins**	**Total phenols**	**DPPH** **(** **IC**_**50**_ **)**
***C. dactylon extract***	+	-	+	-	++	-	**39.82 mg** **/** **g**	**0.70 mg** **/** **ml**

### Effects of the aqueous extract of C. dactylon on angiogenesis in granulation tissue

**Figure 1:**
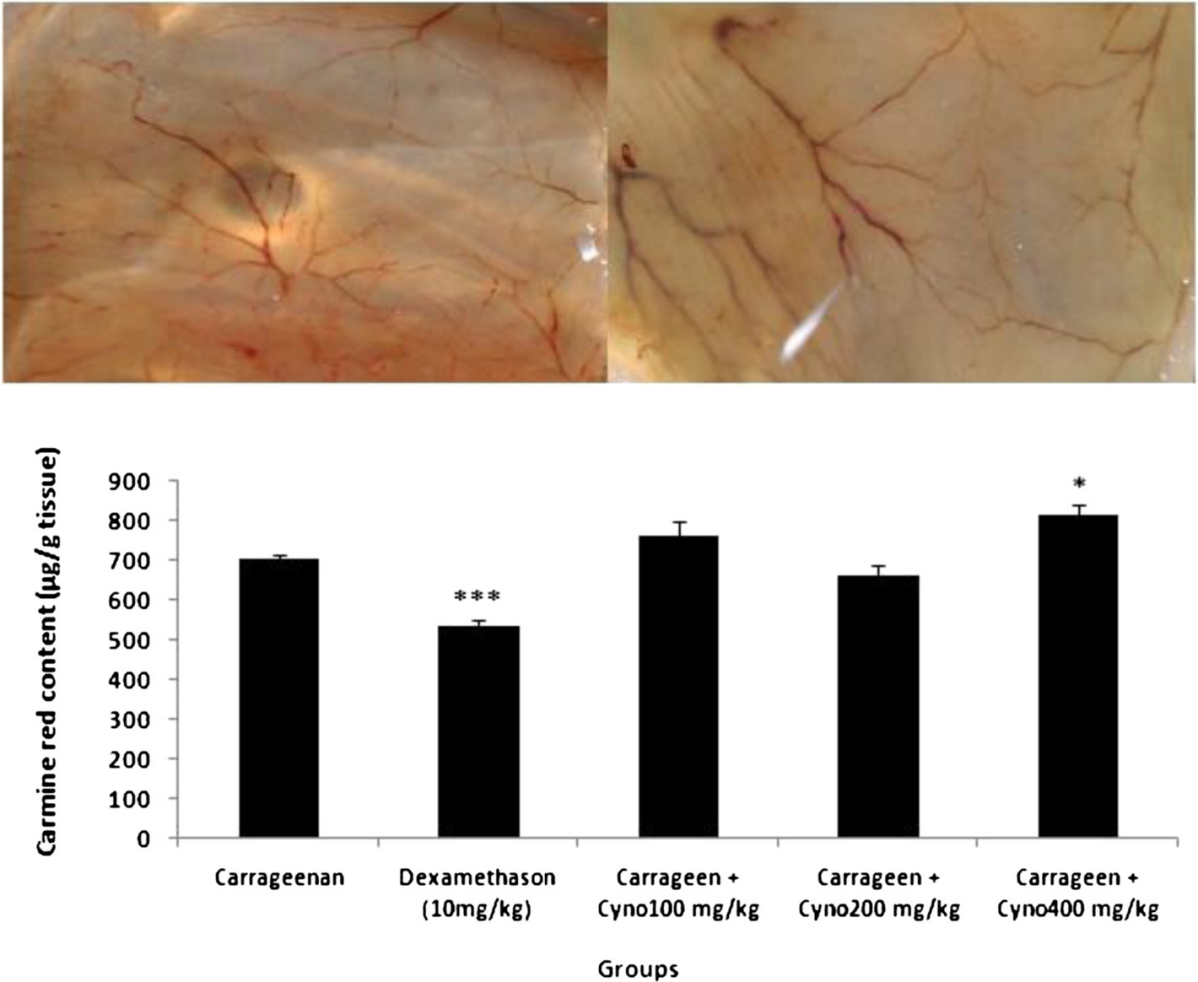
**Upper trace** **:** **Effects of the aqueous extract of** ***C. dactylon*** **(400 mg** **/** **kg) on angiogenesis in granulation tissue versus positive control (carrageenan** **;** **left) in the air pouch model of angiogenesis in rats.** Lower trace: The effect of oral administration of aqueous extract of *C. dactylon* on carmine dye content (as an index of angiogenesis) in granulation tissue in the air pouch model of angiogenesis in rats. Cyno: Cynodon dactylon, Carrageen: Carrageenan. Data represented as mean ± SEM. N = 6. *P < 0.05 and ***P < 0.001 *vs* control group (Carrageenan).

Six days after the injection of carrageenan solution into the air-pouch, a dissectible granulation tissue was formed in the subcutaneous tissue. Following intravenous injection of carmine red dye to the anaesthetized animals, the dye was accumulated in the granulation tissue and the amount of the dye was assessed as an index of angiogenesis. As shown in Figure 1, oral administration of the aqueous extract of *C. dactylon* (400 mg/kg) produced a significant (*P* < 0.05) increase in angiogenesis. In agreement with these findings, vascular network formation was also stimulated by *C. dactylon* as shown in Figure 1 (upper trace; right).

### Effects of the aqueous extract of C. dactylon on pouch fluid volume, leukocyte Infiltration and granulation tissue weight

**Figure 2:**
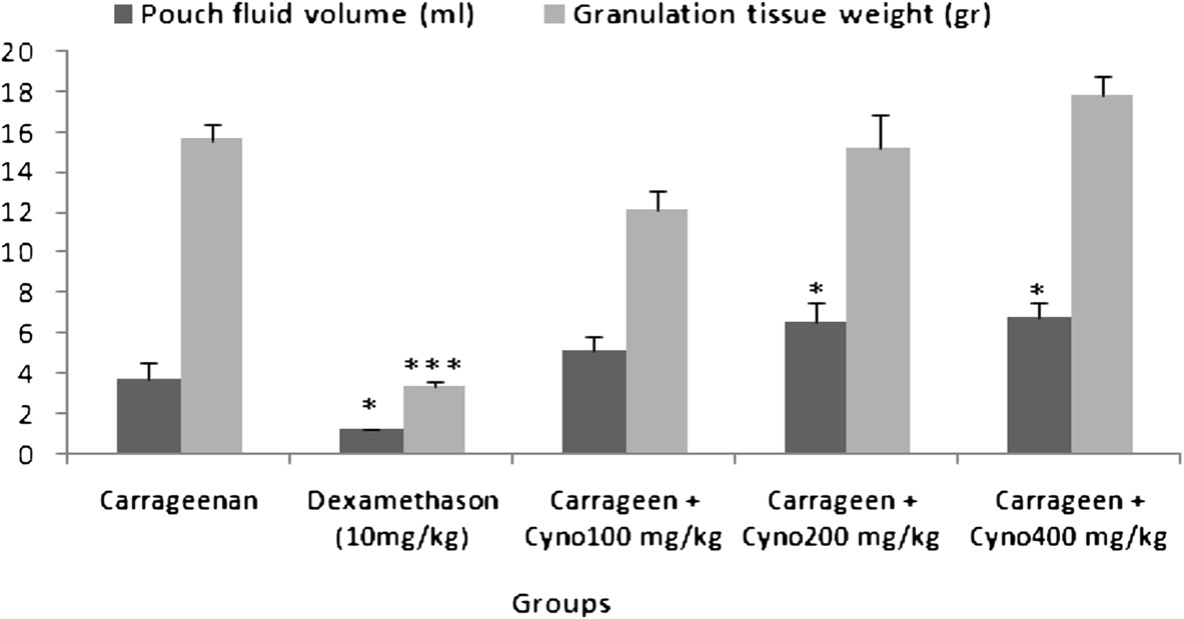
**Effect of the aqueous extract of** ***C. dactylon*** **at various doses on pouch fluid volume and granulation tissue weight 6 days after carrageenan injection.** Cyno: Cynodon dactylon, Carrageen: Carrageenan. Data represented as mean ± SEM. N = 6–8. *p < 0.05 and ***p < 0.001 versus the carrageenan group.

The treatment of rats with oral administration of the aqueous extract of *C. dactylon* at doses of 100, 200 and 400 mg/kg which was started one day before the intra-pouch injection of carrageenan and continued for 6 days, dose dependently reduced neutrophil (p < 0.05) and total leukocyte (p < 0.001) recruitment whereas increased lymphocyte recruitment into the exudates (Table 2). In contrast, administration of *C. dactylon* dose dependently increased pouch fluid volume and granulation tissue weight in comparison to the carrageenan group (Figure 2). Dexamethasone was used as a positive control which significantly reduced total leukocyte accumulation in the exudate, pouch fluid volume and granulation tissue weight compared to the carrageenan group (Table 2, Figure 2).

**Table 2:** **Effect of the aqueous extract of** ***C. dactylon*** **on leukocytes recruitment into the pouch exudate**

	**Carrageenan**	**Dexamethasone**	**Carrageen** **+** **Cyno 100 mg**/**kg**	**Carrageen** **+** **Cyno 200 mg**/**kg**	**Carrageen** **+** **Cyno 400 mg**/**kg**
**Neutrophil percentage**	20 ± 1	12 ± 0.33*	22 ± 1.2	17 ± 3	13 ± 2*
**Lymphocyte percentage**	58 ± 2.8	50 ± 1.8	57 ± .32	59 ± 4.4	67 ± 3.7
**Total leukocyte** **(** **10** ^**5**^ **)**	895 ± 6.4	498 ± 15.2***	635 ± 14.3***	629 ± 33***	523 ± 19.1***

Data represented as mean ± SEM. N = 6. *P < 0.05 and *** *P* < 0.001 *vs* control group (Carrageenan) using one way ANOVA with Student-Newman-Keuls *post*-*hoc* test. Carrageen: Carrageenan; Cyno: Cynodon dactylon.

### Effects of the aqueous extract of C. Dactylon on VEGF expression in human umbilical vein endothelial cells (HUVEC)

**Figure 3:**
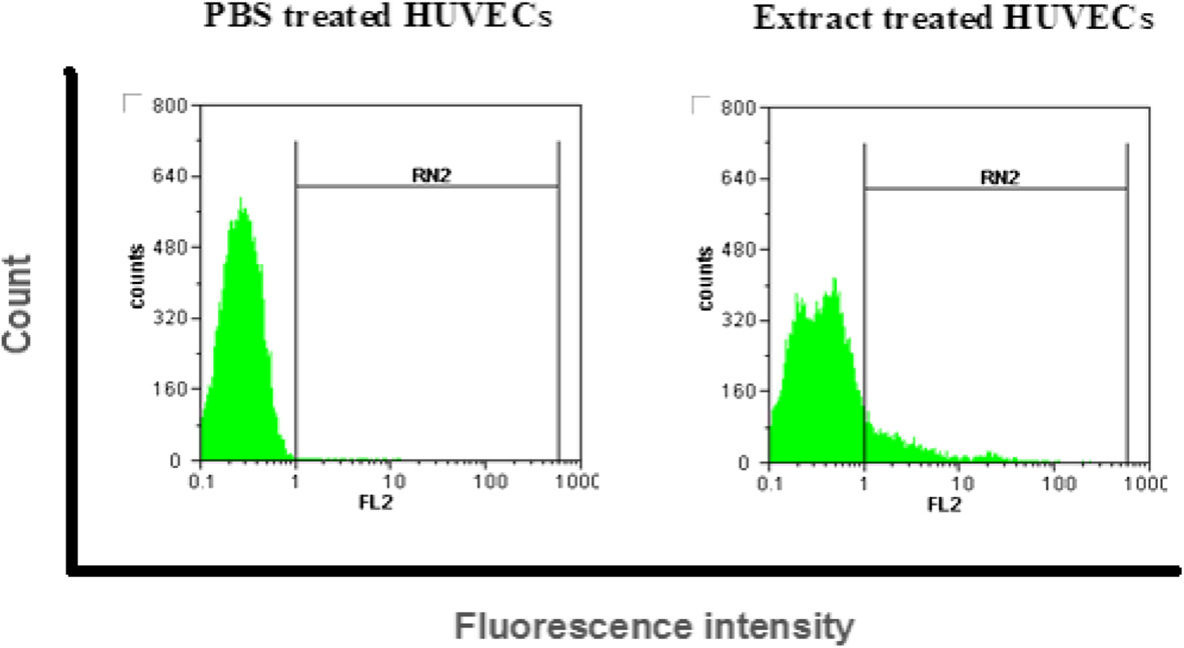
**The expression change of VEGF in HUVECs by flow cytometric procedure.** The expression level of VEGF in PBS treated cells set as control and the expression changes following treatment with *C. dactylon* extract compared to it. The increased expression of VEGF in extract treated HUVECs has been shown as compared to the PBS treated ones. Histograms are representative of three separate experiments.

The results obtained from flow cytometric analysis showed the increased expression of total VEGF (both in cytoplasmic and surface levels) in HUVECs treated with the aqueous extract of *C. dactylon* as compared to those treated with PBS. Three experiments were performed and 12% increase in expression of VEGF was detected in extract-treated cells as compared to the PBS-treated ones (Figure 3).

## Discussion

Overall, as far as we know, the long history of use and prevailing reputation of many types of natural resources, particularly higher plant species, among the nations are impressive. More recently, herbal medicines have been identified as sources of various phytochemicals, many of which possess different countless activities. Accordingly, *C. dactylon* signifying to have medicinally valuable secondary metabolite types of phytochemicals like alkaloids, coumarins, saponins and some types of phenolics might have a role in angiogenesis.

Nonetheless, further research is required to meet the challenges of isolation and structural elucidation of the major active compounds in the plant for identifying an efficient natural medicine which is reliant on a better understanding of the association between chemical constituents and biological properties of natural resources. In this time of increasing requisite for effective, affordable health promotion and treatment strategies for our growing populations and enlarging health problems, the history and reputation of herbal medicines must be examined in a rigorous and scientific way which may be translated into clinical benefit.

*C. dactylon* has been widely used in the traditional medicine of Iran and other countries for the treatment of several cardiovascular conditions such as heart failure [2], and arrhythmias [14]. Moreover, various pharmacological effects of the plant have been proven in several studies [27]. Examples of these effects include analgesic and antipyretic [28], negative ionotropic and negative chronotropic effects [29], anti-arthritic, and anti-inflammatory activities [30]. Although promotion of angiogenesis has been a therapeutic strategy in the treatment of cardiovascular diseases such as ischemic heart disease; however, it can be part of the pathogenesis of several other diseases such as cancer [9,10].

The present study was carried out to evaluate the possible effects of *C. dactylon* on angiogenesis which thought to mediate the part of beneficial effects of the plant in cardiovascular conditions. Accordingly, the possible angiogenic effect of the plant was assessed in both Carrageenan-induced air-pouch model as an *in vivo* and human umbilical vein endothelial cells as an *in vitro* models. The results demonstrated that the constituents present in the aquatic extract of the plant have the potential to increase angiogenesis probably through up-regulation of VEGF-gene expression. Other angiogenesis parameters including exudate volume and granulation tissue weight were increased dose dependently following the administration of the extract. These results were accompanied with the decreased total leukocyte and neutrophil infiltration into the air-pouch. Since decreasing total leukocyte and neutrophil count indicates the anti-inflammatory effect of *C. dactylon* the obtained results were parallel to the findings of the previous studies [7]. However, the extract of *C. dactylon* exerts an anti-inflammatory effect against acute inflammation, while increasing angiogenesis. It has been already shown that simultaneous administration of two different substances may upregulate the angiogenic responses, while downregulating the inflammatory responses [31]. The same effect can be also attributed to different constituents present in the aqueous extract of the plant.

*In vitro* study on the effect of *C. dactylon* on the expression of VEGF in human umbilical vein endothelial cells revealed that there might be a positive correlation between the angiogenic effect of the plant and its ability to increase VEGF expression. This might be the possible underlying mechanism through which the plant exerts its effect. It has been also shown in several studies that the extract of *C. dactylon* possesses significant anti-tumor activities [32,33]. As noted earlier, angiogenesis plays a central role in the pathogenesis of neoplastic diseases [34], however the exact mechanism(s) responsible for such effect of the plant is not yet clearly understood. In the mentioned studies, the sole reversal effects of the plant against undesired consequences of tumors [12] and its anti-oxidant properties [16] have been partly attributed to this effect of the plant without adequate evaluation of other mechanisms affecting tumor growth. For instance, none of these studies have evaluated the *in vivo* angiogenesis changes of the tumors affected by the administration of the extract. Based on the findings of the present study which indicate the potential of the plant to promote angiogenesis at high doses, it has to be taken into consideration that the plant might contain unsafe agents which may aid in the growth of the tumor through increasing the tumor angiogenesis. This is probably the first study that reveals an unsafe aspect of this plant in contrast to its beneficial properties previously shown in anti-cancer studies.

## Conclusion

To the best of our knowledge, this is the first study showing the angiogenic property of the aquatic extract of *C. dactylon*. Based on these findings, *C. dactylon* is suggested as a potential source of angiogenic compounds, the effects of which might be attributable to its increasing effect on the expression of VEGF, a growth factor mainly involved in angiogenesis. Accordingly, this plant can be used in the development of novel herbal medicines for the amelioration of the consequences of conditions such as ischemic heart disease, and other cardiovascular complications. Our findings are in contrast with the results of previously conducted studies on the anti-tumor effects of *C. dactylon*, indicating that in addition to its beneficial effect as an anti-cancer source, it might be a toxic agent due to increasing tumor angiogenesis. Therefore, critical care should be taken on the safety of the plant to be introduced as an anti-tumor agent. Indeed, further studies are required to clarify the precise underlying mechanisms and to identify the safe aspects of the plant to be employed as a therapeutic source.
